# High‐Entropy Nanomaterials for Advanced Electrocatalysis

**DOI:** 10.1002/smsc.202200109

**Published:** 2023-04-05

**Authors:** Sol A Lee, Jeewon Bu, Jiwoo Lee, Ho Won Jang

**Affiliations:** ^1^ Department of Materials Science and Engineering Research Institute of Advanced Materials (RIAM) Seoul National University Seoul 08826 South Korea; ^2^ Liquid Sunlight Alliance (LiSA) Department of Applied Physics and Materials Science California Institute of Technology Pasadena CA 91106 USA; ^3^ Advanced Institute of Convergence Technology Seoul National University Suwon 16229 Republic of Korea

**Keywords:** electrocatalysis, energy conversion, high-entropy alloys, high-entropy materials, nanomaterials

## Abstract

High‐entropy alloys refer to near‐equimolar alloys of five or more elements and are receiving attention due to their unique physical and chemical properties. In electrocatalysis, they serve as active sites in multiple elements, favoring the optimized adsorption/desorption property toward the target reaction. High‐entropy nanomaterials (HENMs) are attractive candidates as electrocatalysts by taking advantage of a high surface‐to‐volume ratio and tailored composition. This review begins with the concept of high‐entropy materials and various strategies for designing electrocatalysts. Then, the recent advances in HENMs as electrocatalysts for various applications (water‐splitting reaction, carbon dioxide reduction reaction, alcohol oxidation reaction, etc.) are introduced with their catalytic performances. Finally, based on the current status of HENMs for electrocatalysis, the challenging aspects and the future insight of HENMs for advanced electrocatalysis are discussed and proposed.

## Graphical abstract

High‐entropy nanomaterials (HENMs) are attracting attention in the field of electrocatalysis because of their remarkable advantages. This review introduces the recent development of HENMs for electrocatalysis and various design strategies such as composition control, morphology control, and computational approach. The current challenges of HENMs as electrocatalysts are provided, and perspectives toward advanced electrocatalysis using high‐entropy materials are suggested.

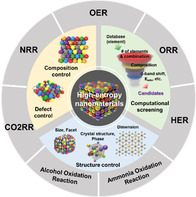

## 1 Introduction

The explosive growth of the world's population causes an increase in energy usage. Therefore, the development of fossil fuels such as oil, coal, and natural gas extends to meet energy demand, raising the Earth's temperature by increasing the CO_2_ emissions.^[^ ^1^ ^]^ As well as accelerating global warming, CO_2_ has a negative effect on humans when exposed for a long time.^[^ ^2^, ^3^ ^]^ Realizing the seriousness of the situation, the Paris Agreement that holds the rise in average global temperature far below 2 °C was signed, and the whole world should cut down the fossil fuel use.^[^ ^4^, ^5^ ^]^ Therefore, research is being conducted to find breakthroughs by developing eco‐friendly energy‐converting applications. Due to their natural abundance, CO_2_, N_2_, and H_2_O are possible sources for producing ideal energy carriers such as alcohol, ammonia, and hydrogen.^[^ ^6^, ^7^, ^8^ ^]^ They are produced by electrocatalytic reactions such as oxygen reduction reaction (ORR), oxygen evolution reaction (OER), hydrogen evolution reaction (HER), CO_2_ reduction reaction (CO2RR), alcohol oxidation reaction, N_2_ reduction reaction (NRR), and ammonia oxidation reaction.

Until now, considerable efforts have been made to catalysts for the high‐performance aforementioned electrocatalytic reactions.^[^ ^9^, ^10^, ^11^, ^12^, ^13^, ^14^, ^15^ ^]^ Monometallic noble metals (e.g., Pt, Pd, Ir, Rh, Ru, and Au) show excellent catalytic performance due to their optimal‐binding energy to reactants.^[^ ^16^ ^]^ To synthesize catalysts with higher performance and superior stability, alloying precious metals with non‐noble transition metals (e.g., Fe, Co, Cu, and Ni) became an alternative.^[^ ^17^ ^]^ Electrocatalysts consisting only of transition metals are recently paid attention due to their affordable costs and abundance.^[^ ^18^ ^]^ Although the non‐noble material‐based electrocatalysts have been developed astonishingly, their catalytic characteristics are insufficient to replace precious metal catalysts.^[^ ^19^ ^]^

To overcome the limitations of conventional catalysts comprising one or two elements, high‐entropy material (HEM) electrocatalysts are in the spotlight.^[^ ^20^ ^]^ The HEM consists of five or more elements, and the concentration of each element is 5–35 at%.^[^ ^21^ ^]^ HEMs have tremendous potential as electrocatalysts due to the synergistic effects of various elements. HEMs show enhanced thermal and catalytic stability resulting from the low Gibbs free energy and the sluggish diffusion effect.^[^ ^22^ ^]^ The lattice distortion caused by the different sizes of the atoms that comprise HEM facilitates the transportation of active species.^[^ ^23^, ^24^ ^]^

In addition, downsizing electrocatalysts to the nanoscale results in improving kinetic characteristics and catalyst properties.^[^ ^25^, ^26^ ^]^ Compared with the bulk catalysts of the same volume, nanoscale catalysts have more active sites, resulting in lower activation energy for the catalytic process.^[^ ^27^ ^]^ When the particle size decreases, new active sites (e.g., steps, edges, corners, and terraces) for catalytic reactions can come into sight.^[^ ^28^ ^]^ As the d‐band center shifts due to catalyst size changes, the electronic configuration may change in favor of the catalytic reaction.^[^ ^29^, ^30^ ^]^

**Figure 1:**
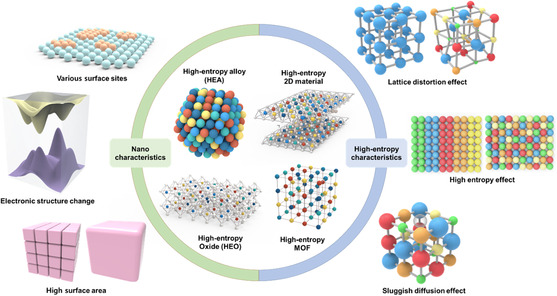
Schematic illustration of various HEMs with their characteristics.

The main purpose of this review is to give an inclusive overview of recent research on various high‐entropy nanomaterial (HENM) electrocatalysts such as high‐entropy alloys (HEAs),^[^ ^31^ ^]^ high‐entropy oxides (HEOs),^[^ ^32^ ^]^ high‐entropy 2D materials,^[^ ^33^ ^]^ and high‐entropy metal‐organic frameworks (HE‐MOFs)^[^ ^34^ ^]^ (**Figure** **1**). First, features and synthesis methods of HEMs will be provided, followed by strategies to design optimized HEM electrocatalysts. Then, this review mainly focuses on applications of nano‐size HEMs for electrocatalysis: ORR, OER, HER, CO2RR, alcohol oxidation reaction, NRR, and ammonia oxidation reaction. Finally, perspectives on the challenges and future developments of HENMs as electrocatalysts are suggested.

## 2 Features and Synthesis of HEMs

### 2.1 Definition

Beyond the traditional metal alloy, the concept of HEA was first proposed by Yeh et al.^[^ ^21^ ^]^ HEA refers to an alloy consisting of at least five elements mixed in an equimolar or nearly equimolar ratio and an amount ranging from 5 to 35 at%.^[^ ^21^ ^]^ In terms of statistical thermodynamics, as the number of elements increases, the increment of mixing entropy is expressed as

(1) $$\Delta S_{\text{mix}}=-R\sum_{i=1}^{n}x_{i}\text{ln}x_{i}\;$$

where $\Delta S_{\text{mix}}$ is the mixing entropy, *R* is the gas constant, $x_{i}$ is the molar concentration of each elemental component, and *n* is the number of principal elements. The maximum value of the entropy is represented by the following expression when the proportions of each component are equal.

(2) $$\Delta S_{\text{mix}}=R\text{ ln }n$$

The value of the entropy depends on the number of elements (*n*). When *n* ≥ 5, mixed entropy becomes $\Delta S_{\mathrm{mix}}\geq \;1.5R$, defined as high entropy.^[^ ^21^, ^35^ ^]^ For $\Delta S_{\text{mix}}<1R$, it is called low entropy and $1R\;\leq \Delta S_{\text{mix}}<1.5R$ for medium entropy.^[^ ^35^ ^]^ Based on the mixing entropy of the material, it can be classified as low‐entropy materials, medium‐entropy materials, and HEMs.

### 2.2 Features

HEMs provide unique characteristics rather than traditional materials because of four core effects: high‐entropy effect, lattice distortion effect, sluggish diffusion effect, and cocktail effect.^[^ ^36^ ^]^ These properties provide HEMs with numerous versatile properties, making them suitable for many applications. In the view of electrocatalysis, the lattice distortion effect enhances the coordination environment of atoms on the catalyst surface or the adsorption energy of intermediates. The high‐entropy effect and sluggish diffusion effect improve the thermodynamic stability of electrocatalysts.^[^ ^37^ ^]^

#### 2.2.1 High‐Entropy Effect

In general, the stability of the phase can be expressed through the Gibbs free energy, and the equation is as follows

(3) $$\Delta G_{\text{mix}}=\;\Delta H_{\text{mix}}-T\Delta S_{\text{mix}}$$

where Δ*G*_mix_, Δ*H*_mix_, and Δ*S*_mix_ are the changes of the Gibbs free energy, mixing enthalpy, and mixing entropy, respectively, and *T* is the thermodynamic temperature. Equation (1) and (2) show that mixing entropy increases proportionally to the number of elements.^[^ ^35^ ^]^ The increment of mixing entropy leads to the low Gibbs free energy, forming a stable single‐phase solid solution structure and the outstanding stability of HEMs.^[^ ^35^, ^38^ ^]^

#### 2.2.2 Lattice Distortion Effect

The lattice distortion effect originates from the difference in the size of elements consisting of HEM. This effect causes severe lattice strain, which contributes to preventing dislocation movement. As a result, the mechanical properties of HEM can be enhanced.^[^ ^39^, ^40^ ^]^ The large difference in the atomic size of elements in HEMs also results in thermodynamic nonequilibrium states, which can contribute to lowering the energy barrier for electrocatalytic reaction (e.g., adsorption of reactant/intermediates and conversion of molecules).^[^ ^20^, ^41^ ^]^ Therefore, the lattice distortion effect makes HEM promising candidates for electrocatalysts with catalytic active sites.^[^ ^35^ ^]^

#### 2.2.3 Sluggish Diffusion Effect

The sluggish diffusion effect occurs due to the distinct potential energy and diffusion rate of various elements.^[^ ^42^ ^]^ Therefore, it slows down the atom diffusion rate and phase transformation speed compared with conventional intermetallic and trimetallic alloys. Interactions between various atoms and lattice distortion affect the diffusion of atoms. Atoms generally diffuse via vacancy mechanisms. The bonding with surrounding atoms changes when an atom moves into a vacancy, which leads to an energy state change.^[^ ^20^ ^]^ If an atom moves to a low‐energy site, it prefers to stay; in the opposite case, it tends to return to its original position. This process suppresses atom diffusion.^[^ ^42^ ^]^ In addition, some elements are less active than others, so it is difficult to fill empty spaces. Phase transformation and grain growth need the coordinated diffusion of various elements.^[^ ^20^ ^]^ The sluggish diffusion effect is important for ensuring the long‐term stability in electrocatalytic performance because it effectively controls the microstructure by reducing the particle growth rates.^[^ ^43^ ^]^

#### 2.2.4 Cocktail Effect

The cocktail effect represents a complex synergistic effect caused by various elements of HEM and is closely related to the characteristics of each element constituting HEM.^[^ ^44^, ^45^ ^]^ Similar to how the density of an alloy decreases when a light element is added, the HEM will have good potential as an electrocatalyst if an active transition metal component is added.^[^ ^46^ ^]^ Therefore, the cocktail effect is a complex effect that is influenced not only by the average properties of constituent elements but also by high entropy, lattice distortion, and slow diffusion.^[^ ^20^ ^]^

### 2.3 Synthesis

There are two types of methods to fabricate HEMs: top‐down methods and bottom‐up synthesis. Although the former is a technology that reduces the size from bulk to nanoscale, the latter is a way to manufacture nanomaterials from atoms and molecules.^[^ ^47^ ^]^ The top‐down method has the advantage of being simple to make large‐scale products, but it is not easy to control the shape and size of the products. The bottom‐up method is possible to create catalysts with uniform sizes and shapes through self‐assembly and growth from reactants.^[^ ^48^, ^49^ ^]^ Several synthesis methods employed for HEMs and their electrocatalytic application are provided in **Table** **1**. In the following subsections, we will discuss various synthesis methods, characteristics, and applications of HEMs.

**Table 1:** Summarized HENMs in different synthesis methods

Method		Material	Morphology	Application	References
Top‐down method	Mechanical alloying	AuAgPtPdCu	NP	CO2RR	[140]
	Dealloying	NiMnFeMo	Nanoporous structure	HER, OER	[141]
	Laser ablation	CoCrFeMnNi	NP	OER	[63]
Bottom‐up method	Sputtering	CrMnFeCoNi	NP	ORR	[67]
	Electrodeposition	CuNiFeCrCo	Nanostructure	HER, OER	[71]
	Hydrothermal synthesis	FeCoNiCuMnN	Nanowire/NS structure	HER	[142]
	Solvothermal synthesis	PdCuMoNiCo	Nano‐hollow spherical structure	ORR	[78]

#### 2.3.1 Top‐Down Methods

**Figure 2:**
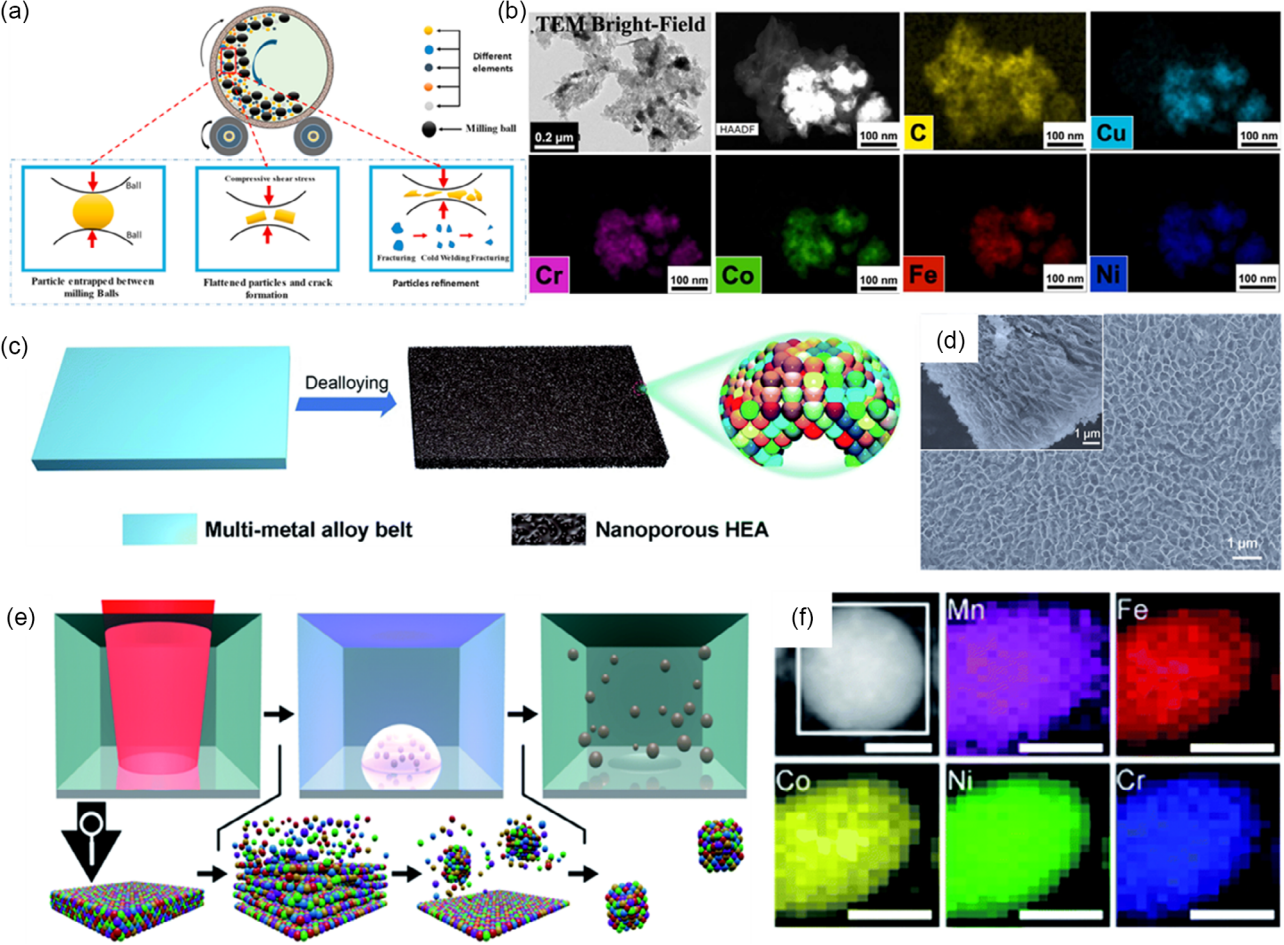
Top‐down method. a) Different stages in mechanical alloying. Reproduced with permission.^[^ ^51^ ^]^ Copyright 2022, Elsevier. b) HAADF‐TEM compositional mapping of NiFeCrCoCu HEA NPs prepared by ball milling. Reproduced with permission.^[^ ^52^ ^]^ Copyright 2022, Elsevier. c) Qualitative representation of the preparation of nanoporous ultra‐HEAs by dealloying. Reproduced with permission.^[^ ^57^ ^]^ Copyright 2021, Royal Society of Chemistry. d) SEM image of the dealloyed‐nanostructured AlNiCoFeMo HEA. Reproduced with permission.^[^ ^58^ ^]^ Copyright 2019, American Chemical Society. e) Schematic illustration of the laser‐based synthesis of HEA NPs. f) SEM image and EDX elemental mapping of laser‐generated CoCrFeNiMn HEA NPs. e,f) Reproduced with permission.^[^ ^63^ ^]^ Copyright 2019, Royal Society of Chemistry.

Mechanical alloying, such as the ball‐milling method, is one of the representative top‐down synthesis methods due to its simplicity. It entails grinding individual powders into mixed powders with the help of controlling agents.^[^ ^50^ ^]^ The fabrication stage of mechanical alloying is shown in **Figure** **2**a.^[^ ^51^ ^]^ Various factors (e.g., milling speed, time, and strength) affect the HEM products during the alloying process. As selected research, Srivastava et al. synthesized NiFeCrCoCu HEA nanoparticle (NP)–graphene composite via mechanical milling for urea oxidation (Figure 2b).^[^ ^52^ ^]^ The mechanical‐milling process reduces the average HEA crystalline size, increasing HEA particle integration into defects in the graphitic layer. Cho et al. reported CuCoNiFeMn HEA by high‐energy ball‐milling (HEBM) process at room temperature.^[^ ^53^ ^]^ The long‐time HEBM process enables uniform distribution of all five elements constituting HEA without noticeable aggregation compared with the short‐time HEBM process. CuCoNiFeMn HEA shows significant bifunctional electrochemical performance for HER and OER in neutral pH. Compared with other materials, the HEA electrode exhibits a smaller overpotential of −320 mV versus reversible hydrogen electrode (RHE) for HER and 80 mV versus RHE for OER.

Dealloying is the method of selectively dissolving specific atoms from multiple metal atomic layers.^[^ ^54^ ^]^ As shown in Figure 2c, the reorganization of remaining atoms and surface modification could be achieved through dealloying.^[^ ^55^, ^56^, ^57^ ^]^ The scanning electron microscopy (SEM) image (Figure 2d) shows that AlNiCoFeMo HEA was obtained from the three‐dimensional nanoporous structure after dealloying Al in an alkaline solution.^[^ ^58^ ^]^ Jiang's group fabricated multiphase‐nanostructured quasi‐eutectic CuAlNiMoFe alloy by dealloying in KOH solution.^[^ ^59^ ^]^ Through this process, HEA ingots became lamellar nanostructure consisting of uniform interpenetrative nanopores. As HER electrocatalysis, this structure provides a high surface area that leads to a low overpotential of ≈56 mV to reach the current density of 100 mA cm^−2^. Similarly, Sun et al. presented AlNiCuPtPdAu HEA NPs by combining the bulk melting, fast cooling, and dealloying.^[^ ^60^ ^]^ During the dealloying, the uniformly distributed second‐level nanoporous structure was formed by removing the pure Al phase and selectively etching the Al_3_(NiCuPtPdAu) alloy compound phase. After the 100 k electrochemical cycles, the catalytic activity of as‐prepared HEA NPs retained 92.5% of its initial property, confirming that the catalyst is one of the most stable Pt‐containing ORR catalysts reported so far.

Pulsed laser ablation is a tool that induces interaction between the solid surface and the intense laser pulse and enables the separation of metal ions from the surface. These ions clump together to make clusters due to electromagnetic forces. Then, NPs are formed and dispersed into the liquid.^[^ ^61^, ^62^ ^]^ As shown in Figure 2e, using the ultrashort‐pulsed laser ablation method, Gokce's group suggested CoCrFeNiMn HEA as an OER catalyst.^[^ ^63^ ^]^ The synthesized NPs were distributed in various sizes, and most of the NPs had a hydrodynamic diameter of 2.8 nm. From the SEM and energy‐dispersive X‐ray spectroscopy (EDX) elemental mapping (Figure 2f), five metal components are uniformly distributed.

#### 2.3.2 Bottom‐Up Methods

The sputtering deposition process is an effective synthetic method for preparing the high density and uniform films and coatings on diverse substrates.^[^ ^64^, ^65^ ^]^ Each target atom is sputtered in a vacuum or controlled gases and is deposited as a thin film on supports.^[^ ^66^ ^]^ Schuhmann and co‐workers reported CrMnFeCoNi as an ORR electrocatalyst using a co‐sputtering system.^[^ ^67^ ^]^ Co‐sputtering using single‐element sputter targets facilitates adjusting the resulting HEA composition by controlling different deposition rates of individual sputter targets. The linear sweep voltammogram (LSV) curve showed a similar overpotential with Pt due to the synergistic effect of five elements. Similarly, Ting et al. prepared the uniform FeNiMoCrAl HEA thin film on Ni foam with the sputter deposition, presenting ORR activity with a low overpotential of 220 mV at 10 mA cm^−2^ and long‐term stability for 50 h.^[^ ^68^ ^]^

**Figure 3:**
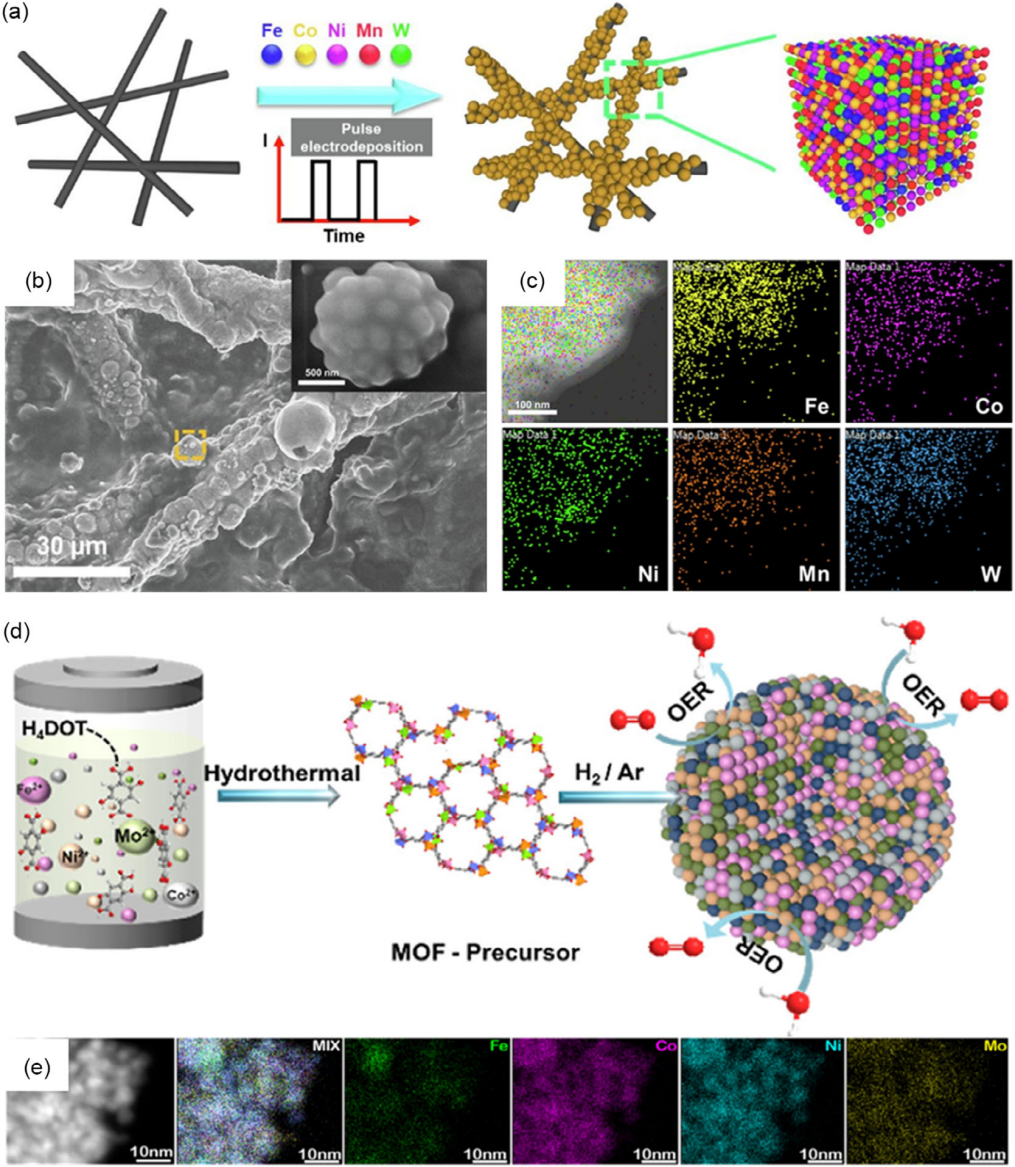
Bottom‐up method. a) Schematic illustration of the pulse current electrodeposition method. b) SEM image of the electrodeposited FeCoNiMnW HEA. c) TEM image and EDX elemental mapping of FeCoNiMnW HEA. a–c) Reproduced with permission.^[^ ^74^ ^]^ Copyright 2022, Elsevier. d) Qualitative representation of the hydrothermal synthesis method and application of FeCoNiMo HEA/C. e) HAADF–STEM–EDS elemental mappings of HEAs. d,e) Reproduced with permission.^[^ ^76^ ^]^ Copyright 2022, American Chemical Society.

Electrodeposition is the conventional and the simplest coating process because it enables composition and morphology change under ambient conditions.^[^ ^69^ ^]^ When a voltage is applied, materials are deposited on the substrates through the redox reaction of metal ions in the electrolyte.^[^ ^70^ ^]^ Simchi's group deposited copper‐rich Cu–Ni–Fe–Cr–Co alloy on Ni foam as HER and OER catalysts.^[^ ^71^ ^]^ Due to the densely packed nanosheet (NS) array with a large surface area and rich electroactive sites, the HEA catalysts showed low onset potentials of 70 mV for the HER and 240 mV for the OER. Pulse electrodeposition is an exceptional method of applying the potential or current with discrete values.^[^ ^72^ ^]^ Pulse electrodeposition can increase the purity and homogeneity of the electrodeposited material.^[^ ^73^ ^]^ As shown in **Figure** **3**a, Lu et al. synthesized high‐entropy FeCoNiMnW alloy on carbon paper with the pulse current electrodeposition.^[^ ^74^ ^]^ SEM image and TEM–EDX elemental mapping are shown in Figure 3b,c, respectively, which demonstrate that the FeCoNiMnW alloy is uniformly dispersed on the substrate. This HEA alloy worked as the electrocatalyst for both HER and OER in acidic media.

Hydrothermal synthesis is a well‐established method to fabricate homogeneous and pure final products at a low cost. In a typical hydrothermal process, precursors dissolve and recrystallize in a closed system, such as an autoclave, under high‐temperature and high‐pressure conditions.^[^ ^48^, ^75^ ^]^ Through the one‐step hydrothermal method, as shown in Figure 3d, Hu et al. suggested FeCoNiMo MOFs/C displays a uniform NP morphology with a particle size of 8 ± 0.3 nm.^[^ ^76^ ^]^ High‐angle annular dark‐field scanning transmission electron microscopy (HAADF‐STEM)–energy‐dispersive X‐ray spectroscopy (EDS) image in Figure 3e shows the uniform dispersion of Fe, Ni, Co, and Mo elements. The solvothermal method is similar to the hydrothermal method, but it uses other substances as solvents not water.^[^ ^77^ ^]^ Hu's group developed PdCuMoNiCo nano‐hollow spherical HEA on a carbon hybrid of reduced graphene oxide (RGO) and carbon nanotubes (CNT) with a ratio of 3:1 (RGO3‐CNTs) (PdCuMoNiCo nano‐hollow spherical (NHS)/RGO3‐CNT) using a one‐pot solvothermal method.^[^ ^78^ ^]^ It has been found that PdCuMoNiCo NHSs/RGO3‐CNT shows longer durability in 0.1 m HClO_4_ for ORR and the best formic acid oxidization performance among Pd‐based catalysts published so far.

### 2.4 Strategies to Design HEM Electrocatalysts

**Figure 4:**
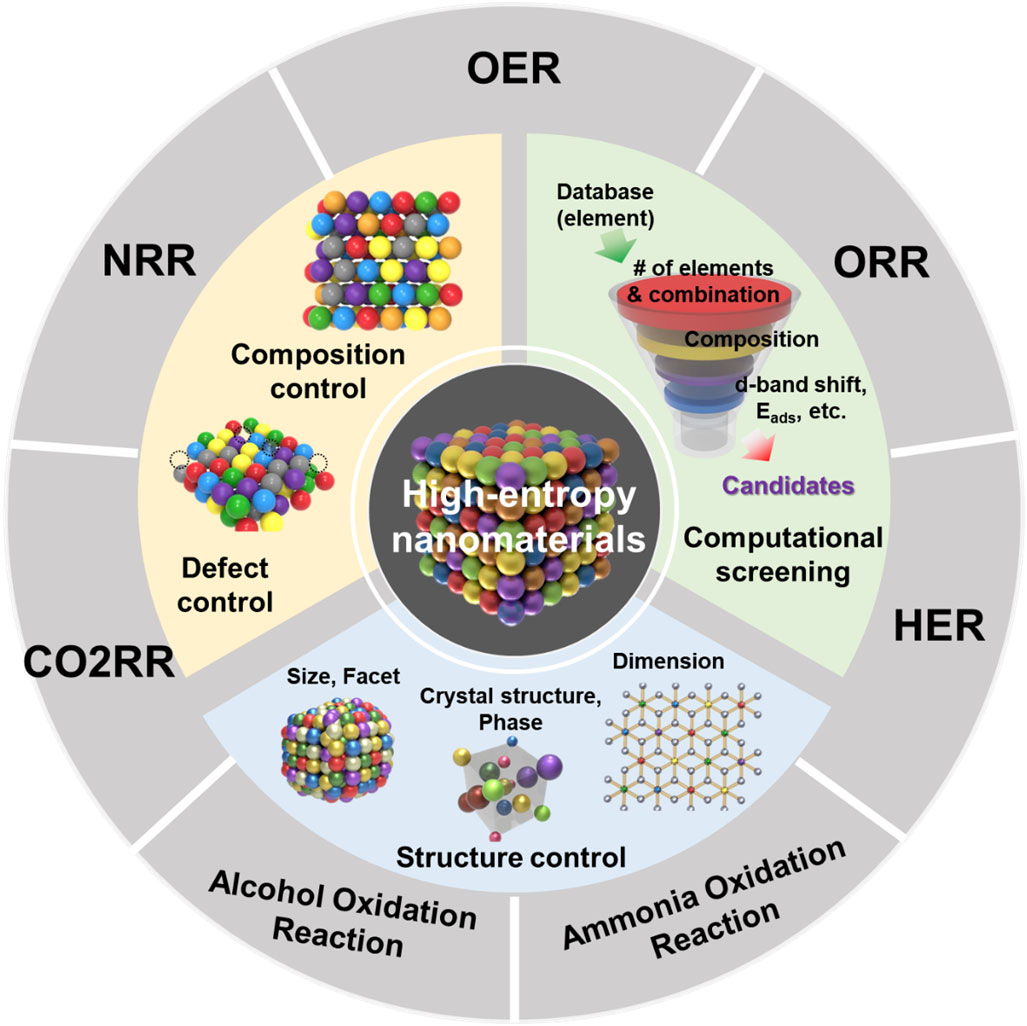
Strategies for developing HENMs for various electrocatalysis.

The representative pathway to enhance the catalytic performance of electrocatalysts is increasing the intrinsic activity of the active sites of the catalysts and the number of active sites.^[^ ^79^ ^]^ The HEMs are promising for efficient electrocatalysts with tunable composition, element combination, and phase. The representative strategies for designing the HEM electrocatalysts are computational screening, composition/defect control, and structure control (size, facet, crystal structure, phase, and dimension), as shown in **Figure** **4**.

HEA, exceeding the traditional alloy with two or three elements, has become a new concept of alloys. The system becomes equilibrium when the Gibbs free energy approaches the minimum value at constant temperature and pressure, as shown in Equation (3). As the number of elements (*n*) increases, the $\Delta S_{\text{mix}}$ becomes maximum Rlnn under equal element composition. Therefore, the Gibbs free energy becomes lower and makes the solution phase stable. HEAs are prone to form a solid solution with simple structures (face‐centered cubic (fcc), body‐centered cubic, or mixed structure).^[^ ^80^, ^81^ ^]^ It should be considered whether the predicted HEAs can be synthesized experimentally. Therefore, the research has been conducted based on thermodynamic calculations to figure out the role of each element on phase stability. This approach serves as a guideline to develop HEAs with phase stability by understanding the phase transition behavior.

HEMs have five or more elements with tremendous possible composition and serve as catalytic active sites for electrocatalysis. Therefore, the theoretical calculations provide the pathway toward rational screening of materials and composition design. The binding energy of the adsorbates can be a descriptor for catalytic activity because of the scaling relations. According to the Sabatier principle, the adsorption energies for key intermediates should have moderate strength, which should neither strong nor weak, to achieve high catalytic activity. Adjusting the adsorption energy of the intermediates of the catalyst surface during the reaction provides information on selectivity and catalytic activity.^[^ ^82^ ^]^ As HEMs can have many material and composition combinations, it is inevitable to screen the predictably effective catalyst candidates and figure out the effects of elements in the reaction pathway rather than empirical approaches.

The composition control of materials can enhance the intrinsic activity of the electrocatalysts by adjusting the active sites of each element. The improvement of the activity of electrocatalysts by alloying with two or three metals has been proven widely. Computation approaches have shown how alloying affects the properties of catalyst activity. For example, Nørskov and co‐workers demonstrated the high‐throughput screening of over 700 binary surface alloys toward HER.^[^ ^83^ ^]^ The catalytic activity of predicted bimetallic alloys as HER catalysts showed good agreement with the computational screening results. They also showed that the relationship between the d‐band center and the chemisorption behavior of bimetallic alloy catalysts results in a volcano‐type curve, which provides information on effective catalysts toward the reaction.^[^ ^84^ ^]^ Experimental reports have shown that controlling the alloy composition has been adjusted for various electrocatalysis. Depending on the target products, various material combinations (e.g., Cu–Au, Cu–Sn, Cu–Zn, and Au–Pd) are used as alloy electrocatalysts for CO_2_ reduction.^[^ ^85^ ^]^ Bimetallic or trimetallic transition metal‐based alloys/(oxy)hydroxides/oxides have been utilized for water splitting and lead to high catalytic activity.

Similar to the traditional metal alloy catalysts, the composition control of HEAs leads to enhanced catalytic activity by the cocktail effect of HEAs. Using computational approaches, Ludwig and co‐workers reported the high‐dimensional composition maps of exemplary Ru–Rh–Pd–Ir–Pt HEA system.^[^ ^86^ ^]^ The activity maps of quinary systems were driven by permutations of six materials libraries. The optimized composition of Ru_25_Rh_15_Pd_31_Ir_15_Pt_14_ was derived by high‐throughput electrocatalytic measurements. The experimental and computational activity of ORR by shape matching of LSVs showed deviation due to the variation of theoretical surface composition. Therefore, leveraging the experimental–computational approach to control the composition of HEMs is a promising direction for designing the active HEM electrocatalysts.

The defects in the crystal structure are formed because of the incorrect structural arrangement of elements. The type of defects can be divided depending on the dimension (D): 0D defects (doping and vacancy), 1D defects (dislocation), 2D defects (twin crystal, stacking fault, grain boundaries, etc.), and 3D defects.^[^ ^87^ ^]^ Defect engineering is one of the promising strategies for controlling the electronic structure and adsorption/desorption sites of materials. In the view of electrocatalysis, defect engineering can affect the catalytic activity by tuning the adsorption behavior of key intermediates. For example, Wei et al. demonstrated the defect‐rich (FeCoNi)_3_O_4−*x*_ crystals by acid‐etching treatment of (FeCoNi)_80_P_14_B_6_.^[^ ^87^ ^]^ The presence of lattice distortion, vacancy defects, and discontinuous fringes of FeCoNiPB/(FeCoNi)_3_O_4−*x*_ leads to the high catalytic activity toward OER with the low overpotentials of 229 mV to reach the current density of 10 mA cm^−2^. Ashwini et al. controlled the number of defects by controlling the metal‐to‐graphene weight ratio (50:50, 70:30, and 90:10) and showed that NiFeCrCoCu/graphene nanocomposite with optimum defect density leads to enhanced urea oxidation performance.^[^ ^52^ ^]^ HENMs will likely have defects during the synthesis process to form a uniform alloy with various elements involved. By controlling the defect formation and defect density of HEMs, therefore, it is possible to regulate the catalytic activity and stability of the electrocatalytic reaction.

Engineering catalyst morphology has been one of the major strategies for developing efficient electrocatalysts by increasing the number of active sites. The main advantages of controlling the morphology of catalysts are: 1) increased surface area for reaction; 2) controlling catalytic active facet; and 3) exposing catalytic active sites. The early development of HEMs was dominated by bulk‐type material synthesis and used for applications that require structural resistance, corrosion resistance, and high‐temperature operation.^[^ ^88^ ^]^ However, the development of synthetic approaches has made HEMs possible from bulk to the nanoscale, which is promising for various catalytic reactions.

Similar to conventional nanomaterials, the HENMs exhibit a high surface area over volume ratio and distinctive surface adsorption energy compared with bulk HEM because of the size effect. Therefore, nanoscale HEMs (e.g., NPs, nanoporous structures, 2D structured materials, and nanostructured HEMs) have been getting attention with the development of advanced synthetic approaches. With the aid of an advanced synthesis method, the size, crystallinity, and shape control of HEA NPs as 0D materials can lead to the tuning of activity and durability of the electrocatalytic properties.^[^ ^89^ ^]^ As a case of shape control, Chen et al. synthesized convex cube‐shaped Pt_34_Fe_5_Ni_20_Cu_31_Mo_9_Ru HEAs toward water splitting and ORR.^[^ ^89^ ^]^ Compared with the cube‐shaped HEAs, the Ru contents in high‐index facets of the convex cube shape and synergistic effects raised by HEAs lead to enhanced catalytic activity. The design of nanoporous HEAs was reported by Qui et al. through the dealloying method.^[^ ^58^ ^]^ Quinary nanoporous AlNiCoFeX (X = Mo, Nb, Cr) showed the highest activity toward OER by taking advantage of nanostructures with increased surface area and chemical composition control. Using a similar synthetic approach, nanoporous AlNiCoIrMo HEAs showed enhanced catalytic durability and high activity toward OER.^[^ ^90^ ^]^ Amorphous Fe_29_Co_27_Ni_23_Si_9_B_12_ HEA ribbons exhibited more favorable OER activity after forming micro/nanopits by electrochemical corrosion. By comparing the crystalline HEA and amorphous HEAs, amorphous HEAs showed the favorable adsorption energy of intermediates, and morphology control of HEA surface enabled exposure of increased active sites toward OER. Hexagonal‐close‐packed (hcp) PtRhBiSnSb high‐entropy nanoplates prepared by the wet‐chemistry method showed high mass activities and durability toward methanol oxidation reaction (MOR), ethanol oxidation reaction (EOR), and glycerol oxidation reaction (GOR).^[^ ^91^ ^]^ The introduction of Rh enhanced the electron transfer and electroactivity in hcp HEA nanoplates.

In nanoscale HEMs, the materials span from crystalline metallic alloys to sulfides, phosphates, oxides, metallic glasses, etc.^[^ ^92^ ^]^ For example, Zhang et al. demonstrated the Co–Cu–Fe–Ag–Mo (oxy)hydroxide HEMs for OER electrocatalysts driven by density functional theory (DFT) calculations and unraveled that introducing electron donor can enlarge the metal–oxygen d‐p hybridization, leading to enhanced electrocatalytic activity.^[^ ^93^ ^]^ High‐entropy Co–Zn–Cd–Cu–Mn sulfide nanoarrays were synthesized on carbon fiber (CF) by cation exchange reaction.^[^ ^94^ ^]^ The CoZnCdCuMnS@CF showed high catalytic activity and durability toward the overall water‐splitting reaction by forming the strong bonding between CF and CoZnCdCuMnS. Glasscott et al. demonstrated the high‐entropy metallic glasses (HEMGs) NPs using nanodroplet‐mediated electrodeposition.^[^ ^95^ ^]^ The HEMG‐NPs consisting of up to eight components were successfully synthesized with precise stoichiometry. Optimized CoFeLaNiPt HEMG‐NPs provided the electrocatalytic sites toward HER and OER with high catalytic activity and stability.

HENMs can be an ideal catalyst by providing diverse adsorption sites and tunable adsorption energy for intermediates for advanced electrolysis. The following section will discuss adjusting appropriate strategies to design HENMs and their effects on catalytic properties.

## 3 Applications of HEMs for Electrocatalysis

HENMs are attracting attention as novel electrocatalysts with improved catalytic activity because of their thermodynamic stability, numerous element combinations, and potentially more active sites for the adsorption of reactants. Compared with conventional binary or ternary nanomaterials, the electronic structures of HENMs can be regulated due to the lattice distortion effects. However, the research of HENMs as electrocatalysis is in its nascent stage. There is plenty of room for exploring the HENM electrocatalysis, including catalytic mechanism study, unveiling active sites depending on the element coordination, and novel strategies to control the properties of HENMs (e.g., facet, morphology, phase, size, and composition) with uniformity. We focused on the recent research of HENMs for advanced electrocatalysis: ORR, OER, HER, CO2RR, alcohol oxidation reaction, NRR, and ammonia oxidation reaction. The catalytic activity of HENM for electrocatalysis and the underlying mechanisms are discussed, and the effects of HENMs with adjusted strategies toward electrocatalysis are discussed.

### 3.1 Oxygen Reduction Reaction

ORR can be divided into four proton–electron transfer reactions and two proton–electron transfer reactions.^[^ ^96^ ^]^ The four proton–electron transfer reactions reduce oxygen into water in an acidic electrolyte (O_2_ + 4 H^+^ + 4e^− ^→ 2H_2_O) and produce hydroxide ions in an alkaline electrolyte (O_2_ + 2H_2_O + 4e^− ^→ 4OH^−^). The two proton–electron transfer reactions generate hydrogen peroxide in the acidic electrolyte (O_2_ + 2 H^+^ + 2e^− ^→ H_2_O_2_) and peroxide ions in the alkaline electrolyte (O_2_ + H_2_O^+^ + 2e^− ^→ HO_2_^−^ + OH^−^).^[^ ^96^, ^97^, ^98^ ^]^

It is well‐known that Pt and Pd are the best catalysts for ORR.^[^ ^99^ ^]^ However, they are expensive, rare, and require an overpotential of 0.3–0.4 V.^[^ ^100^ ^]^ For excellent ORR catalysts, a catalyst should have a fast electrochemical reaction rate, a low overpotential, and cost effectiveness. For several decades, scientists have been searching for electrocatalysts to replace noble metals such as Pt and Pd. HEA has been discovered to be a remarkable candidate for an ORR electrocatalyst.^[^ ^67^, ^101^ ^]^ For instance, Batchelor et al. showed that HEA could be efficient electrocatalysts for ORR by DFT.^[^ ^101^ ^]^ The HEA surface could be designed to maximize catalytic activity by adjusting the composition. It will enhance the number of binding sites with adsorption energies near the volcano curve's peak.

**Figure 5:**
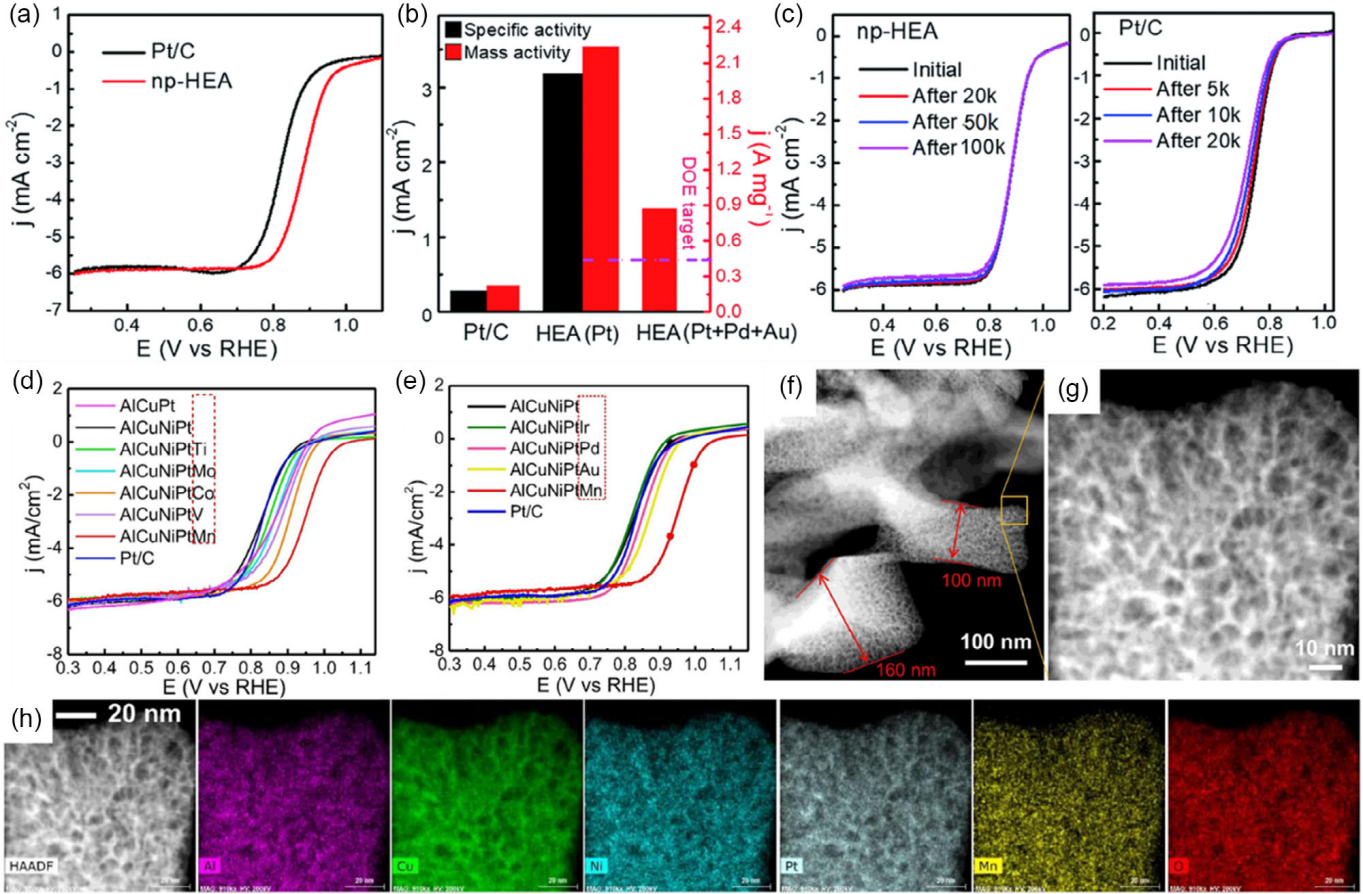
HENMs for ORR. a) ORR polarization curves of HEA and Pt/C. b) Specific activity and mass activities at 0.9 V. c) Stability results after different cycles in O_2_‐saturated 0.1 m HClO_4_. a–c) Reproduced with permission.^[^ ^60^ ^]^ Copyright 2019, Royal Society of Chemistry. d,e) ORR polarization curves of various HEAs. f,g) STEM images of the nanoporous AlCuNiPtMn. h) STEM–EDS mapping of the quinary nanoporous AlCuNiPtMn HEA. d–h) Reproduced with permission.^[^ ^102^ ^]^ Copyright 2020, Elsevier.

There have been several attempts to produce nanoporous HEA alloys with low Pt concentrations. Due to their superior performance compared with commercial Pt/C, researchers suggest that HENMs are expected to be strong candidates for ORR electrocatalysts.^[^ ^60^, ^102^ ^]^ Qui et al. synthesized senary AlNiCuPtPdAu (NP‐HEA), which has a single‐phase nanoporous structure, using a top‐down method (combining bulk melting, fast cooling, and dealloying) instead of the bottom‐up method.^[^ ^60^ ^]^ Using dealloying, they precisely adjusted the composition. The produced HEA was less than 3 nm in size, and a spinel oxide layer was developed on its surface. Due to the spinel oxide layer, this catalyst was stable at temperatures up to 600 °C. Even without an oxide layer, NP‐HEA showed excellent resistance to withstand 10 h at 200 °C due to low diffusivity. As compared with Pt/C (≈0.82 V vs RHE), NP‐HEA showed a significantly positive half‐wave potential shift (≈0.90 V vs RHE), implying remarkably enhanced ORR activity (**Figure** **5**a). NP‐HEA has a specific activity for the electrochemical active surface area of ≈3.2 mA cm^−2^, ≈11.4 times higher than Pt/C (0.28 mA cm^−2^). Despite the small amount of Pt, NP‐HEA showed 10 times more mass activity than Pt/C in the ORR (Figure 5b). In addition, NP‐HEA exhibited endurance and long‐term functional stability in an acidic solution after 100 K cycles, whereas Pt/C showed a 35% loss of mass activity after 20 K cycles (Figure 5c). Using the same top‐down dealloying method, Li et al. synthesized 5‐element nanoporous AlNiCuPtX(X = Pd, Ir, Au, V, Co, Mn, Ti, Mo)HEA, where AlCuNiPt was fixed, and the fifth element was selected among Pd, V, Co, Mn, etc.^[^ ^102^ ^]^ Among the various nanoporous HEAs produced, AlCuNiPtMn HEA, whose fifth element is Mn, revealed ORR catalytic activity and electrochemical cycling durability far superior to Pt/C catalysts. It demonstrated the most‐positive half‐wave potential of ≈0.945 V in an acidic solution compared with ternary NP‐AlCuPt (≈0.86 V) and quaternary NP‐AlCuNiPt (≈0.84 V) (Figure 5d). The catalytic activity was suppressed by the Ni addition in the quaternary alloy AlCuNiPt, which might be owing to the decreased Pt concentration. However, the activity of AlCuNiPt HEA was improved by adding the fifth non‐noble metal to generate quinary HEA (Figure 5d). The addition of noble metal, as the fifth element, increases the quaternary HEAs activity. This effect, though, does not compare favorably to that of non‐noble elements (Figure 5e). The nanoporous HEAs were uniformly mixed into one fcc structure and had the uniform, ultrafine nanopores with a size of 2–3 nm (Figure 5f,g). Ultrafine nanopores increase specific surface area and help mass transfer during catalysis better. The STEM–EDS mapping of the Al, Cu, Ni, Pt, and Mn nanoporous HEA demonstrated how successfully the five elements combine at the nanoscale. Oxygen could also be found because the active elements had been oxidized (Figure 5h). In addition to HEA, there are HEMs with good ORR activity. Hu et al. reported the hollow HE‐MOF containing Mn, Fe, Co, Ni, Cu, and Zn.^[^ ^103^ ^]^ A high‐entropy nanocomposite was prepared by synthesizing through a one‐pot hydrothermal method. Due to metal–particle, metal–alloy, and metal–oxide, which were produced by annealing at 650 °C, it shows good ORR performance. The synergistic effect of the hollow structure and various components is crucial for enhancing the ORR performance. As another example, Li et al. generated HEO NPs that dispersed uniformly on carbon powder substrates through rapid high‐temperature heating.^[^ ^104^ ^]^ It comprises 10 elements (Hf, Zr, La, V, Ce, Ti, Nd, Gd, Y, and Pd). HEO NP demonstrated high stability and enhanced electrocatalytic activity compared with Pd/C.

### 3.2 Oxygen Evolution Reaction

The OER is the half‐anodic reaction in electrochemical water splitting. OER generates oxygen in acid (2H_2_O_(l)_ →O_2(g)_ + 4 H^+^ + 4e^−^) and alkaline (4OH^− ^→ O_2(g)_ + 2H_2_O_(l)_ + 4e^−^) solution.^[^ ^105^ ^]^ HEM is expected to show excellent OER activity because it allows for reasonable element selection and provides many active sites. Therefore, HEM has recently received great attention from researchers as an OER catalyst. **Table** **2** provides the several types of HEM catalysis. OER is a four‐electron reaction in contrast to HER, which is a two‐electron reaction, and the high kinetic energy overpotential slows the reaction rate.^[^ ^106^ ^]^ The electrolysis overpotential and kinetics, which have multiple requirements, limit the OERs efficiency. Ir and Ru metals or their oxides are known as the best OER catalysts.^[^ ^107^, ^108^ ^]^ IrO_2_ and RuO_2_ are efficient catalysts in high demand, but their high cost and scarcity are the main challenge for large‐scale applications.^[^ ^108^ ^]^ Hence, selecting cost effective, catalytic active, and stable materials is required for OER catalysts.

**Table 2:** Summarized HENMs for OER

Material	Substrate	Overpotential [mV at 10 mA cm^−2^]	Tafel slope [mV dec^−1^]	Electrolyte	References
MnFeCoNiCu	Carbon cloth	263	43	1.0 m KOH	[143]
CoFeLaNiPt	HOPGa)	377	150	1.0 m KOH	[95]
AlNiCoFeMo	GCEb)	540	46	1.0 m KOH	[58]
AlNiCoIrMo	GCE	233	55.2	0.5 m H_2_SO_4_	[90]
AlFeCoNiCr	GCE	240	52	1.0 m KOH	[144]
CoCrFeMnNiP	GCE	320	60.8	1.0 m KOH	[145]
K_0.8_Na_0.2_(MgMnFeCoNi)F_3_	GCE	314	55	1.0 m KOH	[146]
FeCoNiCrNb_0.5_	Glassy carbon plate	288	27.7	0.1 m KOH	[147]
FeCoNiSiB	Alloy ribbon	230	85	1.0 m KOH	[148]
MnFeCoNi	CFPc)	302	83.7	1.0 m KOH	[149]
(CrMnFeCoNi)S_*x*_	Carbon substrate	116	66	1.0 m KOH	[33]
CoCrFeMnNi	Ni foam	229	40	1.0 m KOH	[150]
La(CrMnFeCo_2_Ni)O_3_	Ni foam	325	51.2	1.0 m KOH	[151]
AlCrFeNiCu	Ni foam	270	77.5	1.0 m KOH	[152]
AlNiCoRuMo	GCE	245	54.5	1.0 m KOH	[153]
CoCuFeMnNiO	GCE	350	76.7	1.0 m KOH	[154]
(CoNiMnZnFe)_3_O_3.2_	CFP	336	47.5	1.0 m KOH	[155]
(CuNiFeCoZnMnMg)F_2_	Carbon cloth	292	39	1.0 m KOH	[156]
NiCoFeMnCrP	GCE	272	52.5	1.0 m KOH	[118]
NiCoFeZnMo	Ni foam	254 (at 50 mA cm^−2^) 272 (at 100 mA cm^−2^)	61	1.0 m KOH	[34]
Fe_50_Mn_30_Co_10_Cr_10_	Ni foam	247	63	1.0 m KOH	[157]
FeCoNiCuPtIr	MWCNTd) paper	255	61.7	1.0 m KOH	[158]
FeCoNiIrRu	CNFe)	241	154	0.5 m H_2_SO_4_	[159]
FeCoNiMnMo	–	279	56.1	1.0 m KOH	[160]

a) HOPG: highly oriented pyrolytic graphite;

b) GCE: glassy carbon electrode;

c) CFP: CF paper;

d) MWCNT: multiwall carbon nanotube;

e) CNF: carbon nanofiber.

**Figure 6:**
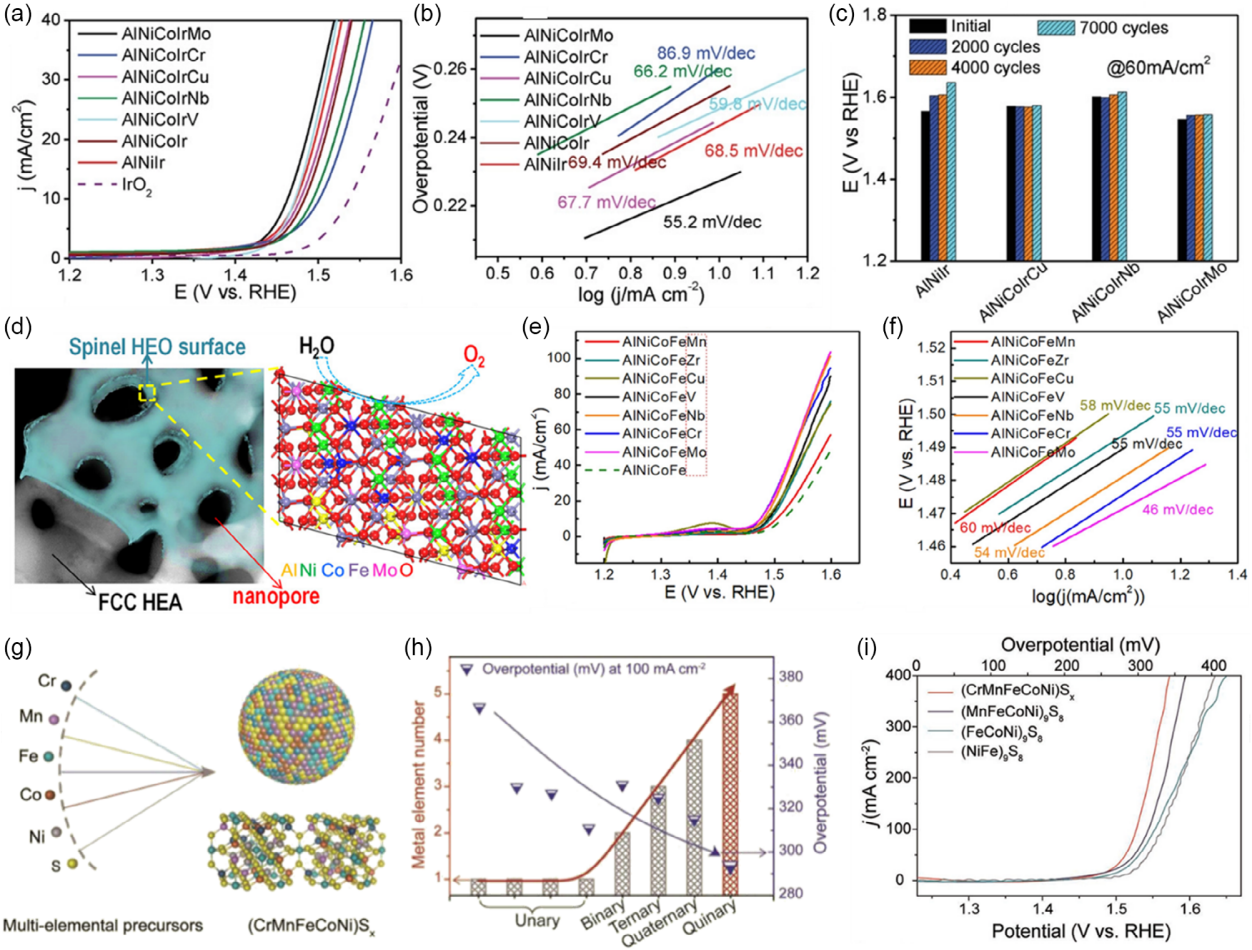
HENMs for OER. a) LSV curves of the various nanoporous HEAs. b) Tafel slope of the various nanoporous HEAs. c) Stability test results after different cyclic voltammetry (CV) cycles in 0.5 m H_2_SO_4_ solution. a–c) Reproduced with permission.^[^ ^90^ ^]^ Copyright 2019, Wiley‐VCH. d) Structure of AlNiCoFeMo HEA covered with oxide. e) LSV curves of the nanoporous HEAs based on AlNiCoFe. f) Tafel slope of the nanoporous HEAs based on AlNiCoFe. d–f) Reproduced with permission.^[^ ^58^ ^]^ Copyright 2019, American Chemical Society. g) Mixing metallic elements (i.e., Cr, Mn, Fe, Co, and Ni) into a high‐entropy sulfide NP. h) Comparison of overpotentials and metal element numbers among unary, binary, ternary, quaternary materials, and quinary HEMS. i) LSV curves of various HES. g–i) Reproduced with permission.^[^ ^33^ ^]^ Copyright 2020, Wiley‐VCH.

Enhancing the effectiveness of electron transport around catalytic centers and the absorption process of the active intermediates formed during the OER are significant considerations for OER catalysts.^[^ ^109^ ^]^ The Ir‐based catalyst can be alloyed with a non‐noble metal to reduce the cost of the catalyst, and the alloy's synergistic effects strengthen the electrocatalytic activity.^[^ ^110^ ^]^ Jin et al. combined Ir with four additional distinct metal elements to synthesize the HEA, AlCoNiIrX (X = Mo, Cu, Cr, V, Nb).^[^ ^90^ ^]^ Mo and V improved OER activity (**Figure** **6**a). DFT calculation indicated that the covalency of Ir—O bonds which was increased by alloying with Ni and X, enhanced catalytic activity. In the case of adding Mo, the covalent bond is stronger and leads to better OER activity. In ternary AlNiIr, the amount of Ir decreases when the fourth and fifth elements are added. However, this has a good impact on the catalytic activity. Even with a small quantity of Ir, HEAs showed improved activity. Especially, AlNiCoIrMo had the best OER activity among various HEAs. It showed an onset potential of 1.42 V versus RHE, the smallest Tafel slope of 55.2 mV dec^−1^, and 233 mV of overpotential (at 10 mA cm^−2^) (Figure 6b). These values are much lower than IrO_2_. The cycling stability in an acidic solution (0.5 m H_2_SO_4_) was excellent, even after 7000 electrochemical cycles (Figure 6c). Even without the presence of noble metals, OER activity can be meaningful. Qui et al. synthesized nanoporous HEAs (AlCoNiFeX(X = Nb, Mo, Cr, V, Zr, Mn, Cu)) with more than five non‐noble metals which are covered with high‐entropy (oxy)hydroxides.^[^ ^58^ ^]^ It was generated by dealloying and chemical etching. The oxidation of the (oxy)hydroxides on the surface is the cause of the broad anodic peaks before the OER (Figure 6d). Among several nanoporous HEAs, AlNiFeCoMo exhibited the best OER activity (Figure 6e). It demonstrated the lowest onset potential (1.44 V vs RHE), 240 mV of overpotential at 10 mA cm^−2^, and the smallest Tafel slope of 46 mV dec^−1^ (Figure 6f). Transition metal sulfides also can be desirable OER catalysts due to their potent catalytic activity. Cui et al. reported high‐entropy metal sulfide (HEMS) for the first time.^[^ ^33^ ^]^ It was produced using the pulse thermal decomposition method to get over the immiscibility of several metallic constituents. Researchers produced quinary (CrMnFeCoNi)S_*x*_ HEMS NPs (Figure 6g). OER activity was measured in 1 m KOH solution. (CrMnFeCoNi)S_*x*_ showed a low overpotential of 295 mV at 100 mA cm^−2^ and excellent stability over 10 h at 100 mA cm^−2^. These values are lower than quaternary and ternary HEMS (Figure 6h,i).

### 3.3 Hydrogen Evolution Reaction

Hydrogen generated by electrochemical water splitting is a promising energy carrier and mitigates environmental pollution and energy crisis. HER undergoes different reaction mechanisms according to the reaction environment. In acidic media, HER occurs by the Volmer–Heyrovsky or Volmer–Tafel step.^[^ ^46^, ^111^, ^112^ ^]^

Volmer step: $H^{+}+e^{-}\to H^{*}$

Heyrovsky step: $H^{*}+H^{+}+e^{-}\to H_{2}$

Tafel step: $H^{*}+H^{*}\to H_{2}$

The electrochemical HER in an alkaline environment involves the reactions of either Volmer–Heyrovsky or Volmer–Tafel step as follows:

Volmer step: $H_{2}O+e^{-}\to H^{*}+{\text{OH}}^{-}$

Heyrovsky step: $H^{*}+H_{2}O+e^{-}\to H_{2}+{\text{OH}}^{-}$

Tafel step: $H^{*}+H^{*}\to H_{2}$

**Figure 7:**
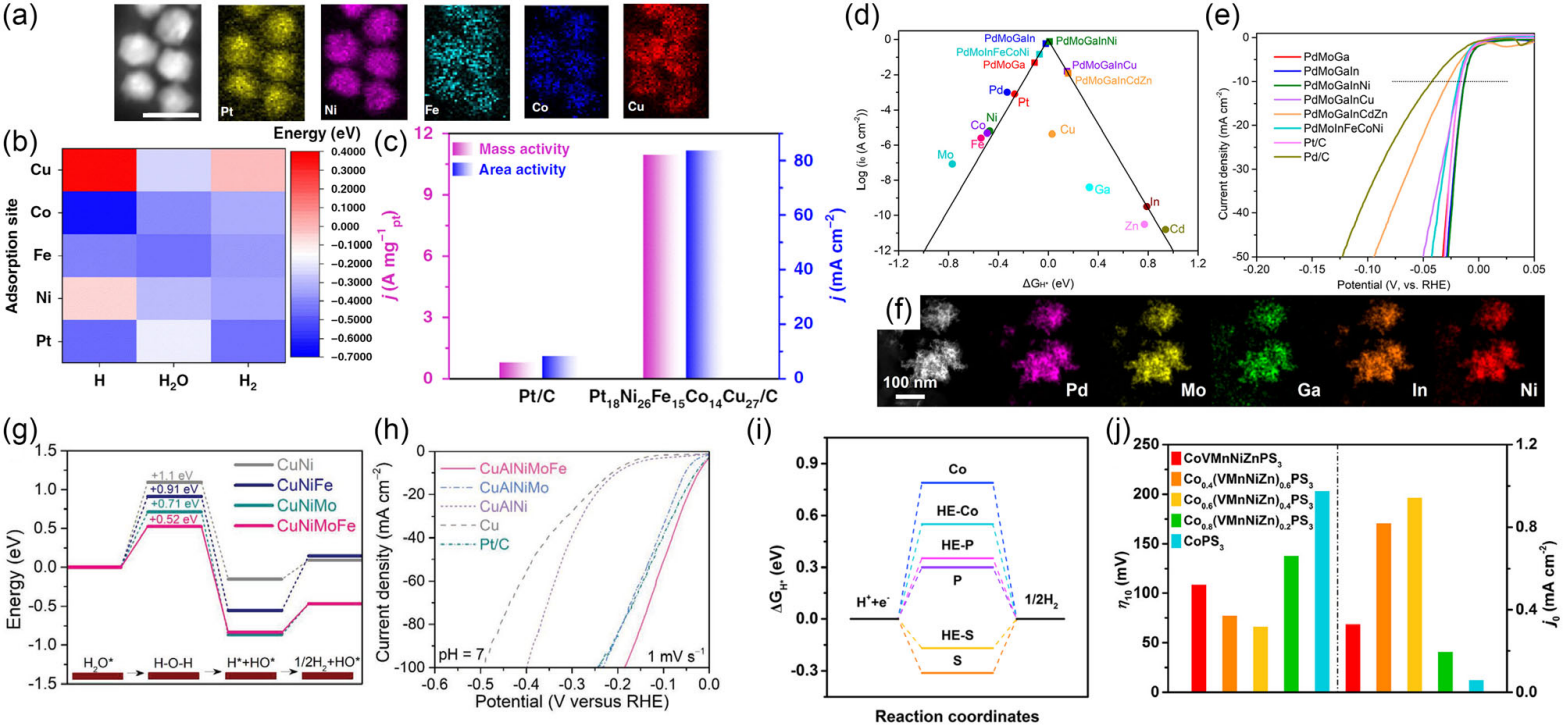
HENMs for HER. a) Elemental mapping of Pt_18_Ni_26_Fe_15_Co_14_Cu_27_ NPs (scale bar: 5 nm). b) Binding energy mapping of HER. c) Comparison of mass activity and area activity for HER at −70 mV versus RHE. a–c) Reproduced with permission.^[^ ^113^ ^]^ Copyright 2020, Springer Nature. d) Volcano plot of HER electrocatalysts achieved by computational method. e) *J–V* curves of HEA electrocatalysts measured in 0.5 m H_2_SO_4_. f) STEM and EDS elemental mapping of HEA PdMoGaInNi NSs. d–f) Reproduced with permission.^[^ ^116^ ^]^ Copyright 2022, American Chemical Society. g) Theoretical calculation of reaction energy for water dissociation. h) Polarization curves of electrocatalysts in 1 m PBS electrolytes. g,h) Reproduced with permission.^[^ ^59^ ^]^ Copyright 2021, Wiley‐VCH. i) Free‐energy diagram of HER at P sites and S sites in the basal‐plane model. j) Overpotentials (*η*_10_) at current densities of 10 mA cm^−2^ and exchange current densities (*j*_0_) of electrocatalysts. i,j) Reproduced with permission.^[^ ^117^ ^]^ Copyright 2022, American Chemical Society.

In general, the Volmer step is a rate‐limiting step, and Pt has been considered as the most active HER catalyst in this reaction. However, Pt is one of the precious metals with costs. Therefore, alloying Pt with additional elements is a promising strategy to reduce the use of Pt and adjust the synergistic effects of multicomponent alloying. Pt‐based HENMs have been developed as outstanding HER electrocatalysts. Li et al. demonstrated high‐entropy Pt_18_Ni_26_Fe_15_Co_14_Cu_27_ NPs as HER and MOR catalysts.^[^ ^113^ ^]^ The PtNiFeCoCu HEA NPs were synthesized by the one‐pot oil‐phase synthesis method. Elemental mapping revealed that each element (Pt, Ni, Fe, Co, and Cu) was uniformly distributed in HEA NPs with a size of ≈3.4 nm (**Figure** **7**a). They conducted DFT calculations to figure out the HER mechanisms at the HEA surface. The adsorption site mapping revealed that the adsorption preference for the intermediates (H, H_2_O, and H_2_) was varied at the HEA surface sites. As shown in Figure 7b, the adsorption of H_2_O occurred at the Fe sites to dissociate water molecules (Volmer step) and stabilize *OH in the hollow sites near Ni and Co. Ni and Co serve as *H adsorption preferred sites to stabilize H in the hollow sites. The overall weak‐binding energy of the generated H_2_ indicates the efficient HER properties of HEA NPs. Figure 7c shows the mass activity of Pt_18_Ni_26_Fe_15_Co_14_Cu_27_/C in comparison with commercial Pt/C. The HEA NPs revealed ≈13.2 times higher mass activity at −0.07 V versus RHE. Similar research was reported by Liu et al. where PtAuPdRhRu alloy NPs were prepared by ultrasonication‐assisted wet chemistry method.^[^ ^114^ ^]^ They prepared ternary PtAuPd, quaternary PtAuPdRh, and PtAuPdRhRu alloy NPs and showed that PtAuPdRhRu HEA NPs/C exceeded HER performance compared with commercial Pt/C in 1.0 m KOH. Furthermore, HEAs with tunable compositions over 10 different elements have been demonstrated. Yu et al. reported nanoporous HEAs comprising 12 different elements for multifunctional electrocatalysis.^[^ ^115^ ^]^ Mn_70_Ni_7.5_Cu_7.5_Co_4.2_V_4.2_Fe_2_Mo_2_Pd_0.5_Pt_0.5_Au_0.5_Ru_0.5_Ir_0.5_ showed low overpotential of 21 mV at 10 mA cm^−2^ to drive HER in alkaline electrolyte. Cai et al. demonstrated nanoporous ultra‐HEAs including 14 elements (Ag, Al, Au, Co, Cu, Fe, Ir, Mo, Ni, Pd, Pt, Rh, Ru, and Ti) as water‐splitting electrocatalysts.^[^ ^57^ ^]^ HEA electrocatalysts showed efficient hydrogen evolution activity by achieving an overpotential of 32 mV at 10 mA cm^−2^ in 0.5 m H_2_SO_4_ electrolytes.

The design of Pt‐based alloys can tune hydrogen‐binding energy, which is closely related to the activity of HER catalysts. Fu et al. predicted the Pt‐free HEAs having optimal hydrogen‐binding energy by a computational method.^[^ ^116^ ^]^ They took Fe, Co, Ni, Cu, Zn, Ga, Mo, Ru, Pd, Ag, Cd, In, Sn, and Au into account for element combinations after preliminary screening. As shown in Figure 7d, they constructed a volcano plot based on the calculated hydrogen‐binding energy in terms of Gibbs free energy and exchange current density derived experimentally. It was figured out that the Pd–Mo–Ga family can be promising materials for HER as an alternative to Pt. PdMoGaIn and PdMoGaInNi with optimal hydrogen‐binding energy were considered as promising HER electrocatalysts. The PdMoGaInNi HEA NSs exhibited better HER activity than the commercial Pd/C and Pt/C electrocatalysts, with a low overpotential of 13 mV to reach 10 mA cm^−2^ (Figure 7e). The homogeneous element distribution of PdMoGaInNi HEA NSs was confirmed through EDS elemental mapping (Figure 7f).

Although the Pt‐based catalysts show low overpotential and a small Tafel slope, Pt is one of the noble metal group elements with a high price, which is inappropriate for the commercialization of H_2_ production. Therefore, efforts have been made to replace noble‐metal‐based catalysts with abundant transition metal‐based catalysts. Yao et al. introduced nanoporous CuAlNiMoFe electrocatalysts for nonacidic HER.^[^ ^59^ ^]^ DFT calculation of HER of CuNi, CuNiFe, CuNiMo, and CuNiMoFe was conducted to figure out the reaction energy variation of HER. As shown in Figure 7g, the energy barrier for rate‐limiting water dissociation decreased from 0.91 eV on the CuNiFe surface to 0.52 eV on the CuNiMoFe surface. It showed that the incorporation of an element affects the absorption of intermediates during HER. The nanoporous CuAlNiMoFe electrode recorded the lowest overpotential of 56 mV to reach the current density of 100 mA cm^−2^ than NiMo of 65 mV, CuAlNiFe of 186 mV, and CuAlNi of 316 mV (Figure 7h).

2D transition metal‐based materials are considered as promising HER electrocatalysts because of their high surface‐to‐volume ratio and catalytic active sites for HER. Wang et al. designed metal phosphorous trichalcogenides (MPCh_3_) as efficient HER catalysts by taking both advantages of layered morphology and high‐entropy strategy.^[^ ^117^ ^]^ DFT calculations were conducted to scrutinize the effects of a high‐entropy strategy for HER activity of MPCh_3_. As shown in Figure 7i, replacing Co with multiple metal atoms affects the positive shifts of Δ*G*_H*_ values in P and S sites, indicating that the S sites at the edge are catalytic active sites for HER. They prepared Co_*x*_(VMnNiZn)_1−*x*_PS_3_ NSs by conventional solid‐state reaction followed by ultrasonication‐assisted exfoliation to confirm the DFT calculation prediction. The *J–V* curves of MPCh_3_ NSs were measured in a 1 m KOH with iR correction to examine HER performance. Optimized Co_0.6_(VMnNiZn)_0.4_PS_3_ NSs showed enhanced HER property with an overpotential of 65.9 mV at the current density of 10 mA cm^−2^ (Figure 7j). Lai et al. proposed transition metal phosphides (NiCoFeMnCrP) NPs as efficient water‐splitting electrocatalysts.^[^ ^118^ ^]^ The phosphorus in phosphides can tune the electronic structure, and metal phosphides usually possess high catalytic activity. The facile control of element incorporation could form NiCoFeMnCrP NPs on the carbon matrix by the sol–gel method. By taking advantage of the high‐entropy effect, NiCoFeMnCrP NPs exhibited outstanding catalytic properties toward HER, OER, and overall water splitting. HER electrocatalysts using HEMs are summarized in **Table** **3**.

**Table 3:** Summarized HENMs for HER

Material		Electrolyte	Tafel slope [mV dec^−1^]	Overpotential [mV at 10 mA cm^−2^]	References
With Pt	Co–Fe–Ni–Pt–Ta–vxc72	0.5 m H_2_SO_4_	37	10.6	[161]
	FeCoNiCu@Pt	1 m KOH	30.6	13.7	[162]
	Pt_34_Fe_5_Ni_20_Cu_31_Mo_9_Ru	1 m KOH	27	20	[89]
	PtAuPdRhRu	1 m KOH	62	–	[114]
	FeCoPdIrPt	1 m KOH	82	42	[163]
	Pt18Ni26Fe15Co14Cu27/C	1 m KOH	30	11	[113]
	NiCuAuPdPtAlO	0.5 m H_2_SO_4_	28	–	[60]
	NiCoFePtRh	0.5 m H_2_SO_4_	30.1	27	[164]
	IrPdPtRhRu	1 m KOH	–	17	[165]
	Mn_70_Ni_7.5_Cu_7.5_Co_4.2_V_4.2_Fe_2_Mo_2_Pd_0.5_Pt_0.5_Au_0.5_Ru_0.5_Ir_0.5_	1 m KOH	29.5	21	[141]
	Al_87_Ag_1_Au_1_Co_1_Cu_1_Fe_1_Ir_1_Mo_1_Ni_1_Pd_1_Pt_1_Rh_1_Ru_1_Ti_1_	0.5 m H_2_SO_4_	30.1	32	[57]
Without Pt	AlNiCoRuMo	1 m KOH	30.3	24.5	[153]
	Ni_20_Fe_20_Mo_10_Co_35_Cr_15_	0.5 m H_2_SO_4_	41	107	[166]
	AlNiCoIrMo	0.5 m H_2_SO_4_	33.2	18.5	[90]
	CuAlNiMoFe	1 m PBS (pH 7)	50	23	[59]
	FeCoNiAlTi	1 m KOH	40.1	88.2	[167]
	NiCoFeMoMn	1 m KOH	29	14	[168]
	PdFeCoNiCu/C	1 m KOH	39	18	[169]
	FeCoNiMnRu/CNFs	1 m KOH	67.4	5	[170]
2D materials	CoCrMnFeNiP	1 m KOH	85.5	136	[145]
	Co_0.6_(VMnNiZn)_0.4_PS_3_	1 m KOH	65.5	65.9	[117]
	CoZnCdCuMnS	1 m KOH	93.4	173	[94]
	NiCoFeMnCrP	1 m KOH	94.5	220	[118]
	PdPtCuNiP	1 m KOH	37.4	32	[171]

### 3.4 Carbon Dioxide Reduction Reaction

Converting CO_2_ into valuable carbon‐based fuels is a promising and sustainable pathway to not only deal with the pollution raised by CO_2_ emission but also offer renewable energy sources.^[^ ^119^ ^]^ Diverse products can be obtained via electrocatalytic CO2RR; C_1_ (CO, CH_4_, HCOOH, HCHO, CH_3_OH), C_2_ (C_2_H_5_OH, CH_3_COOH, C_2_H_4_, etc.), and C_3+_ products.^[^ ^120^ ^]^ It is denoted as C_*x*_ products, where *x* is the number of carbons included in the products. However, CO_2_ is a thermodynamically stable molecule, and a multiple proton–electron coupled transfer process occurs during the CO2RR. At the same time, competitive HER can take place, which hinders achieving high faradaic efficiency (FE) and selectivity of CO2RR.^[^ ^121^, ^122^ ^]^ Designing highly efficient and selective electrocatalysts for CO2RR is imperative. Various strategies, such as doping, defect engineering, alloying, tuning electronic structures, and nanostructuring, have been adjusted to design CO2R electrocatalysts.^[^ ^123^ ^]^ The researchers figured out the selectivity trend of metals toward the CO2RR using calculations.^[^ ^124^ ^]^ Among elements, Cu is the sole pure metal for C_2+_ products.^[^ ^125^, ^126^ ^]^ Cu has been extensively investigated for CO2RR because of its optimal adsorption energy, which leads to high selectivity and catalytic activity.^[^ ^127^ ^]^

**Figure 8:**
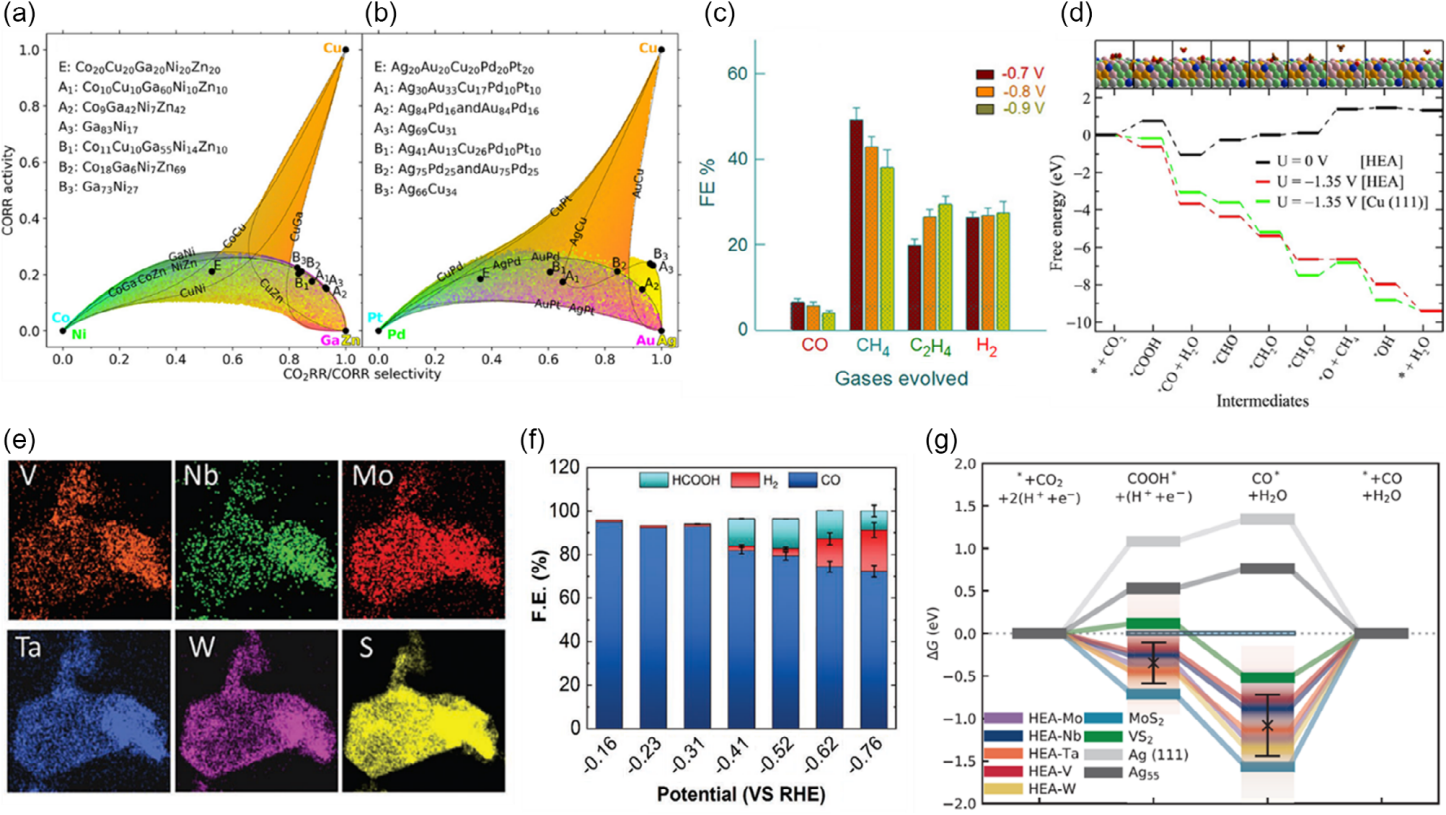
a,b) HENMs for CO2RR. CO2RR/CORR selectivity–CORR activity plots of CoCuGaNiZn (a) and AgAuCuPdPt (b). a,b) Reproduced with permission.^[^ ^128^ ^]^ Copyright 2020, American Chemical Society. c) Faradaic efficiencies of products (CO, CH_4_, C_2_H_4_, and H_2_). d) Free‐energy diagram of CO2RR. c,d) Reproduced with permission.^[^ ^140^ ^]^ Copyright 2020, American Chemical Society. e) EDS mapping of elements TMDC HEA. f) FE of HCOOH, H_2_, and CO of TMDC HEAs at different potentials. g) Theoretical calculation of free‐energy pathway of CO_2_ reduction. e–g) Reproduced with permission.^[^ ^129^ ^]^ Copyright 2021, Wiley‐VCH.

The predicted CO2RR electrocatalysts using HEAs were driven using DFT calculations in combination with machine learning. The catalyst candidates were screened based on the adsorption energy of CO and H, which can imply the selectivity of materials toward CO2RR.^[^ ^128^ ^]^ Selectivity–activity plots of CoCuGaNiZn (**Figure** **8**a) and AgAuCuPdPt (Figure 8b) represented as CO2RR/CORR selectivity and CORR activity space were obtained to predict the catalysts candidates with optimized composition. These plots illustrate that the trade‐off between CO2RR/CORR selectivity and CORR activity can depend on alloy composition; thus, the optimal composition of the HEAs can be driven based on probabilistic access without knowledge of the catalytic activity of elements. Nellaiappan et al. experimentally demonstrated the AuAgPtPdCu HEA alloys as efficient electrocatalysts for CO_2_ to CO conversion and supported the AuAgPtPdCu HEA alloy as outstanding catalysts rather than Cu metal by DFT calculations.^[^ ^128^ ^]^ As shown in Figure 8c, AuAgPtPdCu HEA alloys exhibited the FE of CO, CH_4_, C_2_H_4_, and H_2_ at different applied voltages. Considering that the catalytic activity originates from Cu and other elements contributes to synergistic effects toward CO2RR, HEAs are outstanding electrocatalysts with high hydrocarbon FE. The free‐energy diagram of CO2RR on the AuAgPtPdCu HEA alloys and Cu is shown in Figure 8d. At −1.35 V, the reaction profile showed a downhill form, and Cu showed the uphill curve at *OCH_3_ to *O conversion step. This result indicates that HEAs have thermodynamic advantages for *OCH_3_ destabilization and *O stabilization at the catalyst surface.

Beyond metal alloys, 2D HEMs are getting attention because of their large surface area. Layered 2D high‐entropy transition metal dichalcogenides were demonstrated as electrocatalysts for converting CO_2_ to CO.^[^ ^129^ ^]^ The sulfide alloys were selected by elements having a triangular prismatic 2H phase. (MoWVNbTa)S_2_ nanoflakes were synthesized by the chemical vapor transport method followed by liquid‐phase exfoliation. Elemental mapping exhibited that all elements are well distributed without phase segregation (Figure 8e). The FE of HCOOH, H_2_, and CO at different potentials is shown in Figure 8f. Up to −0.31 V versus RHE, the FE of CO formation was over 90%. When a potential of −0.41 V versus RHE was applied, the FE of CO formation decreased, and HCOOH as a liquid product was observed. The free‐energy pathway of CO_2_ reduction at Mo, W, V, Nb, and Ta is shown in Figure 8g. CO desorption is the rate‐limiting step for the reaction as the reaction goes uphill. From the free‐energy diagram, V and Nb sites have lower CO desorption energy.

### 3.5 Alcohol Oxidation Reaction

**Figure 9:**
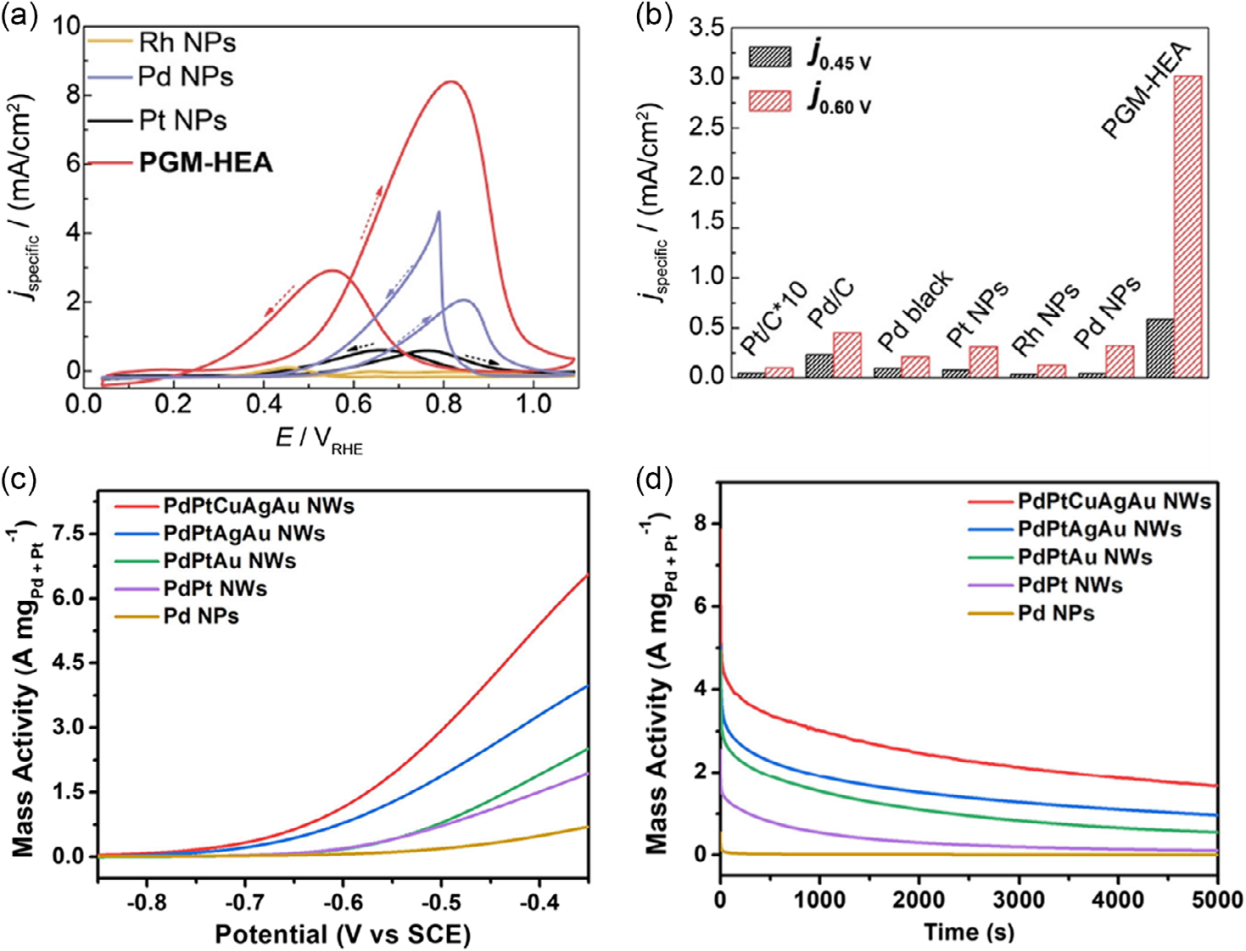
HENMs for alcohol oxidation reaction. a) CV curves of PGM–HEA and monometallic NPs in 1.0 m KOH + 1.0 m ethanol. b) Comparison of the *j*_specific_ values of PGM–HEA at potentials of 0.45 V and 0.6 V in the forward scan with those of the monometallic catalysts and the reported Au@PtIr/C. a,b) Reproduced with permission.^[^ ^133^ ^]^ Copyright 2020, American Chemical Society. c) CV curves of PdPtCuAgAu HEA NWs and other Pd‐based nanomaterials in 1.0 m KOH + 1.0 m ethanol. d) Chronoamperometry curves obtained with PdPtCuAgAu HEA NWs and other Pd‐based nanomaterials in 1.0 m KOH + 1.0 m ethanol. c,d) Reproduced with permission.^[^ ^132^ ^]^ Copyright 2022, Elsevier.

Direct alcohol fuel cells have attracted much attention as alternative energy conversion systems due to their advantages, low‐pollution emission, low cost, and high‐energy density.^[^ ^130^ ^]^ Noble metals such as Pd, Au, and Pt are active catalysts for alcohol oxidation. However, CO, which is produced as byproduct during alcohol oxidation acts as a strong poison that degrades catalytic performances.^[^ ^131^ ^]^ The synergistic effects of HEMs improve the resistance to CO poisoning, leading to higher electrocatalytic stability.^[^ ^132^ ^]^ Pt_18_Ni_26_Fe_15_Co_14_Cu_27_ NPs showing catalytic performance for methanol oxidation were reported by Wang et al. In their work, the Pt_18_Ni_26_Fe_15_Co_14_Cu_27_/C catalyst showed higher stability than commercial Pt/C due to the synergistic effect of five elements.^[^ ^113^ ^]^ Kiragawa and co‐workers applied HEA NPs of all six platinum group metals (PGMs) (denoted as PGM–HEA) for the ethanol oxidation reaction in 1.0 m KOH solution with 1.0 m ethanol.^[^ ^133^ ^]^ PGM–HEA showed a much higher *j*_specific_ value in comparison to single platinum group metal NPs (**Figure** **9**a). Moreover, compared with monometallic catalysts, PGM–HEA showed 2.5–30.1 times higher *j*_specific_ values at 0.45 and 0.60 V in the forward scan (Figure 9b). Xu et al. synthesized ultrathin high‐entropy PdPtCuAgAu nanowires for alcohol oxidation in 1.0 m KOH solution with 1.0 m ethanol.^[^ ^132^ ^]^ As shown in Figure 9c, PdPtCuAgAu nanowires (NWs) showed the largest mass activity and the lowest onset potential among other Pd‐based metal nanocatalysts. In addition, PdPtCuAgAu NWs displayed a long stability of 5000 s with a slow decay (Figure 9d). Yao's group proposed a novel core‐shell structured NHEA@NHEA‐Pd catalyst for the ethanol oxidation reaction.^[^ ^134^ ^]^ NHEA@NHEA‐Pd catalyst is synthesized with a two‐step process: fabricating non‐noble HEA (FeCoNiSn) NPs and surface decoration with Pd. Compared with single‐element Pd, NHEA@NHEA‐Pd exhibits enhanced stability due to the entropy stabilization effect of HEA.

### 3.6 Nitrogen Reduction Reaction and Ammonia Oxidation Reaction

Ammonia is in the spotlight as both a fertilizer and carbon‐free hydrogen carrier.^[^ ^135^, ^136^ ^]^ It is liquefiable at room temperature due to its low volatility and high volumetric density of hydrogen.^[^ ^11^ ^]^ Therefore, electrocatalytic research on making and decomposing ammonia has been actively conducted. Ammonia could be produced through the nitrogen reduction process and decomposed to hydrogen through the ammonia oxidation process. Various active sites on HEMs enhance electrocatalytic performance.^[^ ^137^ ^]^ Wang et al. synthesized uniform RuFeCoNiCu NPs for NRR.^[^ ^138^ ^]^ It is reported that RuFeCoNiCu/CP showed a low overpotential (0.05 V vs RHE) and high FE (38.5%). This catalyst also demonstrated excellent stability during the 100 h chronoamperometry experiment. Hu and co‐workers fabricated PtPdRhRuCe NPs and employed them as the ammonia oxidation catalyst.^[^ ^139^ ^]^ The results showed that the HEA NPs exhibited ≈100% conversion of ammonia and >99% selectivity toward nitrogen oxides such as NO and NO_2_.

## 4 Challenges and Outlook

HEMs have recently attracted attention as promising electrochemical catalysts due to their high catalytic activity based on their unique effects. We focused on the concept of HENMs, including synthetic approaches and introduced various electrochemical reactions (ORR, OER, HER, CO2RR, alcohol oxidation reaction, NRR, and ammonia oxidation reaction) with catalyst design strategies. HENMs provide suitable adsorption energy and more active sites than conventional transition metal catalysts or ternary and quaternary catalysts.

Despite their distinctive properties, there are still fundamental and practical issues of HENMs as electrocatalysts to be considered. First, moderate synthesis strategies for HENMs are needed to be developed. The HEM synthesis method usually occurs in harsh environments (high temperature, high pressure, etc.) to form a single‐phase HEM. It is much more difficult to synthesize nanoscale HEMs with multiple elements. Current research succeeded in developing HENMs without phase segregation. The advanced synthetic approach will produce uniform and tunable HENMs with high yields under mild conditions.

Second, structural control of HENMs is required to enhance the electrocatalytic properties. It is well‐known controlling the catalysts’ size, crystallinity, facet, morphology, and composition significantly impacts their performances. The design of HENMs with distinctive structures is in its infancy; therefore, developing HENMs with tunable structures will lead to advanced electrocatalysis.

Third, the optimization of element combination and composition of the HENMs should be conducted. HENM is a material with five or more elements; thus, it has a variety of possible element combinations. Therefore, screening the HENM candidate materials by computational technique (DFT calculations, machine learning) is required to save time and costs for catalyst development. The research showed that the d‐band center of the alloy and the absorption energy are related to electrocatalysis. It affects the metal–molecule interaction depending on the d‐band center position. Thus, it influences catalytic activity by changing the absorption of key intermediates. The HENM candidates can be derived toward specific target electrolysis using appropriate descriptors.

Fourth, the reaction mechanism of HENMs as electrocatalysis should be figured out in detail. The enhanced catalytic activity of HENMs is explained by the cocktail effect. However, it is an ambiguous concept to verify their scientific effect on catalytic activity in depth. It should be figured out how the catalytic activity of HEMs was improved, and the reaction mechanism process proceeded. With the aid of computational approaches, the adsorption energies and the electronic structures of HENMs can serve as clues to solve the underlying question. In addition, the exposed atoms on catalytic surfaces and surface defects should be carefully examined by advanced characterization technology at an atomic level. Adjusting both computational approaches and materials characterization technology will unravel the mechanism of catalytic enhancement of HENMs.

**Figure 10:**
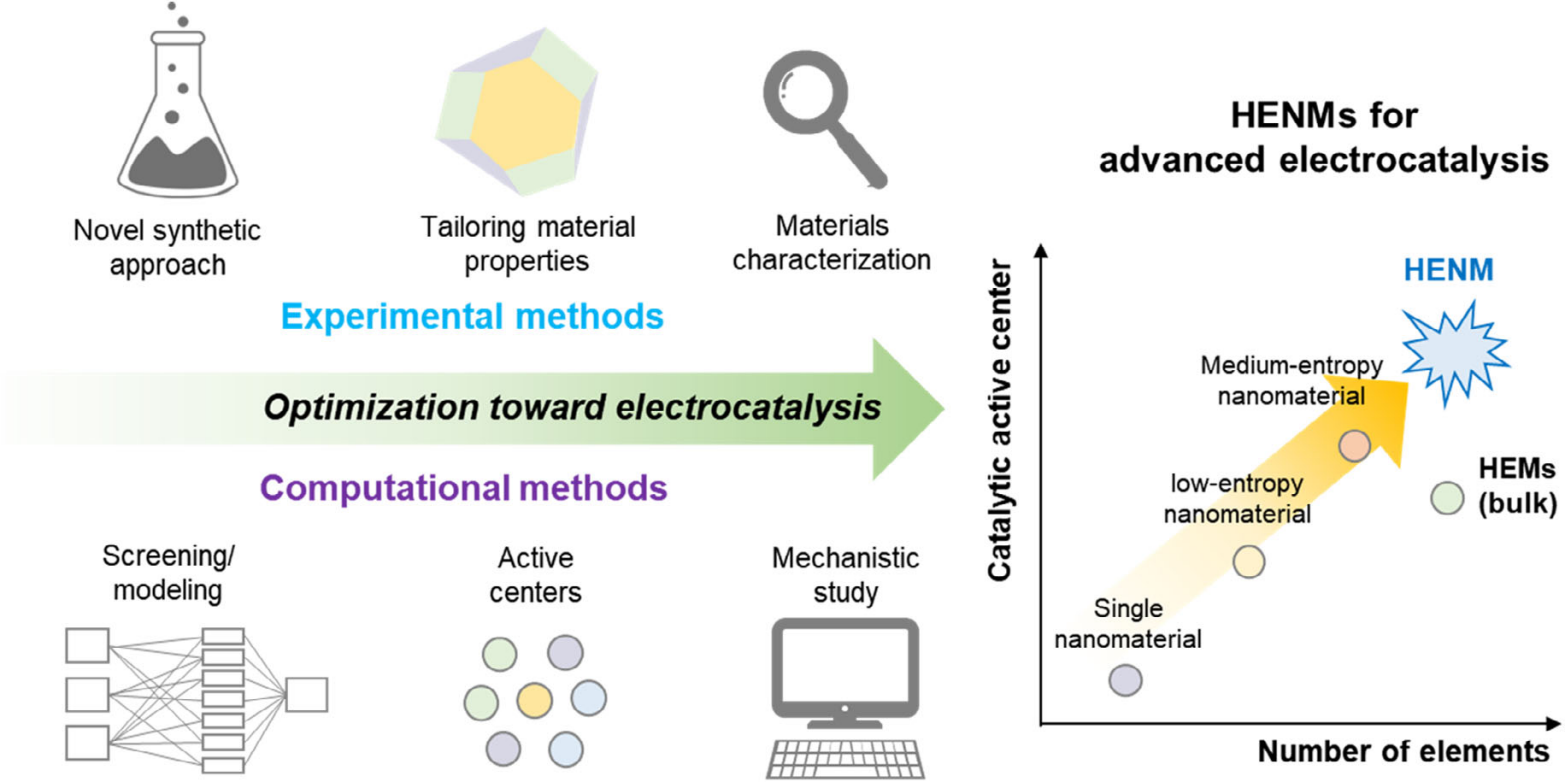
Promising directions of developing HENMs as advanced electrocatalysts.

The research of HENMs has been drastically proceeding in recent few decades and getting attention for advanced electrocatalysis because of their distinctive properties. However, many aspects of HENMs as electrocatalysts are remained to be investigated. **Figure** **10** shows the overall strategies for designing HENMs based on experimental and computational methods. The promising direction toward developing HENMs includes the following aspects: developing an advanced synthetic approach, unveiling the relationship between the properties of HENMs (electronic structures, surface, element composition, etc.) and the catalytic activity, and discovery of reaction mechanisms, including the active sites. It is fascinating that not only can HENMs be used for advanced electrocatalysis but also applied in expanded applications. We hope this review provides an overview of HENMs to researchers and conveys that developing HENMs can be promising new materials for energy conversion applications.

## Conflict of Interest

The authors declare no conflict of interest.
